# Supplementary material for: Cyclodextrin Diethyldithiocarbamate Copper II Inclusion Complexes: A Promising Chemotherapeutic Delivery System against Chemoresistant Triple Negative Breast Cancer Cell Lines
Source: Pharmaceutics. 2021 Jan 10;13(1):84. doi: 10.3390/pharmaceutics13010084 (PMC7826515; doi:10.3390/pharmaceutics13010084)
Supplement: Supplementary file 1 [file pharmaceutics-13-00084-s001.pdf]

pharmaceutics

Supplementary Materials: Cyclodextrin Diethyldithiocarbamate Copper II Inclusion Complexes: A Promising Chemotherapeutic Delivery System against Chemoresistant Triple Negative Breast Cancer Cell Lines

Ammar Said Suliman [1], Mouhamad Khoder [2], Ibrahim Tolaymat [1], Matt Webster [1], Raid G. Alany [2], Weiguang Wang [3], Abdelbary Elhissi [4] and Mohammad Najlah [1,*]

1.1. UV Spectrophotometer Analytical Method Validation

1.1. Methods

The analytical method was validated as per ICH guidelines (ICH Q2 (R1), 2005), which includes selection of the wavelength, linearity, limit of detection, limit of quantification, accuracy, repeatability, intra-day and inter-day precision tests. The drug was dissolved in DMSO to make 0.5 mg/mL as stock solution. The dilutions with DMSO were performed to make a solution with 17 mg/L concentration. The resulting solution was scanned in UV spectrophotometer (Evolution TM 220 series with INSIGHT TM software, Thermo Fisher Scientific) between 220 and 500 nm wavelength. The selected wavelength was at 435 nm. Regarding linearity, different aliquots of DDC-Cu in DMSO were prepared from the same stock solution ranging from 0.6 to 40.2 mg/L and the aliquots absorbance at 435 nm was plotted versus the concentrations. The sensitivity of measurements of DDC-Cu was estimated in terms of the limit of quantification (LOQ) and limit of detection (LOD). The LOQ and LOD were calculated based on the standard deviation of the response and the slope (ICH Q2 (R1), 2005), using equation $LOD = 3.3 \times N/B$ and $LOQ = 10 \times N/B$, where 'N' is standard deviation of the intercept of the calibration curve of the drugs around the noise, and 'B' is the slope of the corresponding calibration curve. For the accuracy determination, a known amount of standard stock solution was added at 80%, 100% and 120% levels to pre-analysed sample solution, and the recovery were determined using the same calibration curve. In terms of repeatability, it was determined by analysing 30 mg/L concentration of DDC-Cu solution for six times. Finally, the precision of the method was performed as intraday and inter-day variations. Intraday precision was determined by analysing the 20, 25 and 30 mg/L of DDC-Cu solutions for three times in the same day. Intra-day precision was determined by analysing the 20, 25, and 30 mg/L of DDC-Cu solutions daily for 3 days over the period of week.

1.2. Results

Three peaks can be assigned from Figure (A.1) which represents the absorbance of solutions of DDC-Cu with different concentrations at wavelengths ranging between 250–500 nm. The highest absorbance value was recorded at 272 nm. Other peaks were observed at 290 and 435 nm. The selected wavelength was at 435 nm because it reflects the presence of copper ions. A strong linear relationship over the concentration range 0.6–49.4 mg/L for DDC-Cu is noticed from the data representing the calibration curve. Linear regression equation was found to be $Y = 0.0265X - 0.0025$ with a high regression coefficient ($R^2 = 0.9997$). The result is presented in Table (A.1). The 10% *w/w* HP-CD and SBE-CD solutions were selected for the accuracy study. The results of recovery that reported in Table 1 showed that the percentage amount found was over 99.0% with % RSD > 2. The percentage relative standard deviation (% RSD) represents the precision of the developed analytical method. These results show reproducibility of the assay because the % RSD values are less than 2, which indicates an acceptable level of precision for the determination of the drug. The LOD and LOQ for DDC-Cu, which were predicted from the calibration curve equation, are 0.06 mg/L and 0.188 mg/L, respectively. The %RSD of the repeatability test at 100 mg/L drug concentration was less than 1.0, whereas the %RSD of the ruggedness test for the same drug concentration is less than 2.0.

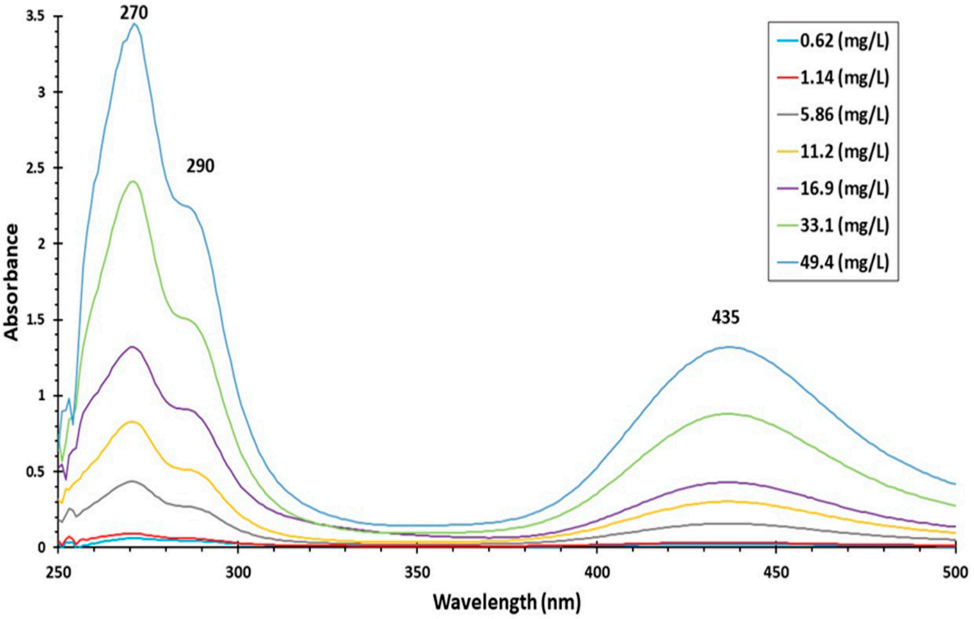

Figure S1. Overlain UV spectra of DDC-Cu from 250 to 500 nm wavelengths.

Table S1. Linearity, recovery, precision, repeatability and ruggedness studies of DDC-Cu.

**Linearity**	**Concentration (mg/L)**	**Absorbance (mean ± SD) (n = 5)**		**%RSD**	***R*²**
	0.62	0.018 ± 0.001		2.87	0.9997
	1.14	0.032 ± 0.001		2.83	
	5.86	0.155 ± 0.001		0.45	
	11.2	0.300 ± 0.001		0.45	
	16.9	0.427 ± 0.001		0.20	
	25.3	0.667 ± 0.001		0.19	
	33.1	0.876 ± 0.001		0.17	
	40.2	1.062 ± 0.002		0.16	
	49.4	1.312 ± 0.003		0.21	
**Recovery**	**Pre-analysed Sample Solution (mg/L)**	**Amount Drug Added (mg/L) (n = 3)**	**Amount Recovered (mg/L) (n = 3)**	**% Recovery**	**%RSD**

10 % HP-CD	254	000.0	253.2	99.69	1.1
		203.2	456.6	99.87	0.3
		254.0	505.1	99.43	0.6
		304.8	556.3	99.55	0.4
10% SBE-CD	257	000.0	255.9	99.57	1.2
		205.6	461.7	99.81	0.5
		257.0	512.8	99.77	0.2
		308.4	563.7	99.70	0.7
**Precision**	**Concentration (mg/L)**	**Intraday Precision (n = 3)**		**Interday Precision (n = 3)**	
		Conc. Found	%RSD	Conc. Found	%RSD
	100	99.60	1.57	99.90	1.67
	150	148.9	0.77	149.2	1.35
	200	199.4	1.23	199.3	0.79
**Repeatability**	**Amount Taken (mg/L)**	**Amount Found (mean ± SD) (n = 6)**		**%RSD**	
	100	99.10 ± 0.27		0.272	
**Ruggedness**	**Amount Taken (mg/L)**	**Amount Found (mean ± SD)**			
		**Analyst I**		**Analyst II**	
	100	99.07 ± 1.3	98.6 ± 0.88		

2. DDC-Cu Intrinsic Solubility Prediction in Water

2.1. Methods

The intrinsic solubility of DDC-Cu in water was determined by using DMSO as co-solvent. This was due to the limit of detection of the drug by UV analysis when measured directly using shake flask method. An excess amount of DDC-Cu was added to 1 ml of DMSO/water mixtures (different ratios ranging between 70 and 90 %*v*/*v* of DMSO). The resulting solutions were vortexed and sonicated at room temperature for 120 minutes before they were agitated for 3

days at room temperature by Stuart reciprocating shaker SSL2 at 250 rpm. After that, the solutions were centrifuged using a centrifuge rotor (Thermo Scientific Heraeus PIC017) for 10 minutes at 13,000 rpm. The supernatants were transferred to Spin X centrifuge tube filter 0.45 μm cellulose acetate in 2.0 mL polypropylene tube from Coster for filtration under the same centrifugation conditions. Triplicate samples were produced to ensure reproducibility of the results.

2.2. Results

The relationship between DDC-Cu solubility in DMSO/water mixtures and the percentage of DMSO in these mixtures are exponential (Figure (B.1)). A similar relationship was demonstrated in case of hydrophobic drug, cinnarizine, with attempt to measure its apparent solubility in phosphate buffer with methanol (Vithlani, Sarraf, and Chaw, 2012). The intrinsic solubility can be predicted from the exponential equation, which is equal to the value of the intercept. In other words, the intrinsic solubility of DDC-Cu in water is 700 ng/L.

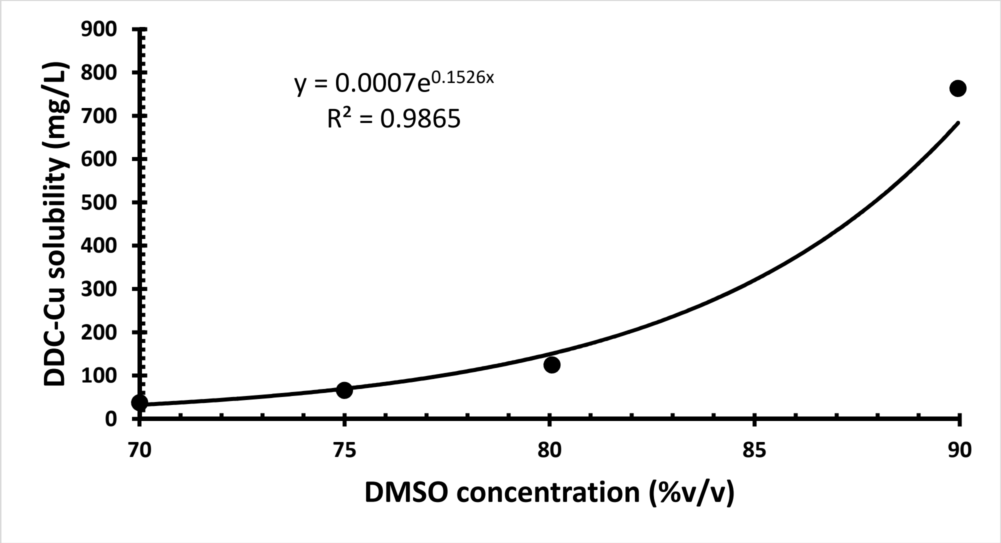

Figure S2. Solubilization of DDC-Cu in DMSO/water mixtures plotted against DMSO concentrations (%*v*/*v*).

3. pH Measurements

The pH values of the cyclodextrin solutions and DDC-Cu/CDs Solutions after 3-day of shaking were measured using a Jenway 3510 pH meter (Cole-Parmer, UK).

Table S2. pH values of the CDs solutions and saturated DDC-Cu/CDs Solutions.

Names	CDs Solutions	Saturated DDC-Cu/CDs Solutions
1% SBE-CD	6.51 ± 0.56	5.65 ± 0.31
5 % SBE-CD	5.59 ± 0.47	5.94 ± 0.21
10% SBE-CD	5.30 ± 0.67	5.95 ± 0.48
15% SBE-CD	5.15 ± 0.46	6.05 ± 0.76
20% SBE-CD	5.12 ± 0.59	6.49 ± 0.43
1% HP-CD	6.79 ± 0.47	5.35 ± 0.58
5% HP-CD	6.70 ± 0.42	5.28 ± 0.66
10% HP-CD	6.38 ± 0.32	5.40 ± 0.38
15% HP-CD	6.13 ± 0.49	5.45 ± 0.85
20% HP-CD	5.91 ± 0.56	5.41 ± 0.94
